# Supplementary figures and images for: Appropriate Imaging Modality for the Etiologic Diagnosis of Congenital Single-Sided Deafness in Children
Source: J Clin Med. 2018 Dec 4;7(12):515. doi: 10.3390/jcm7120515 (PMC6306910; doi:10.3390/jcm7120515)

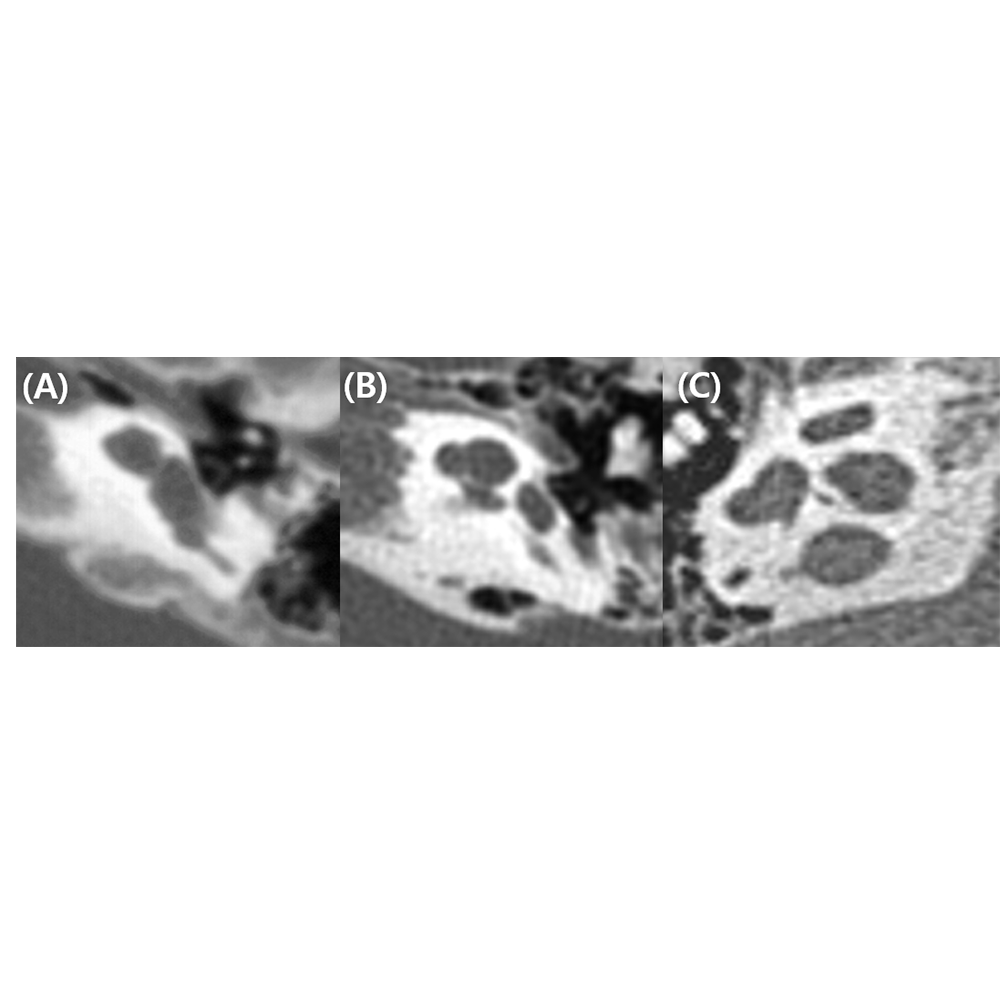

Supplement: Supplementary file 1 [file jcm-07-00515-s001.zip › Supplement 2.tif]

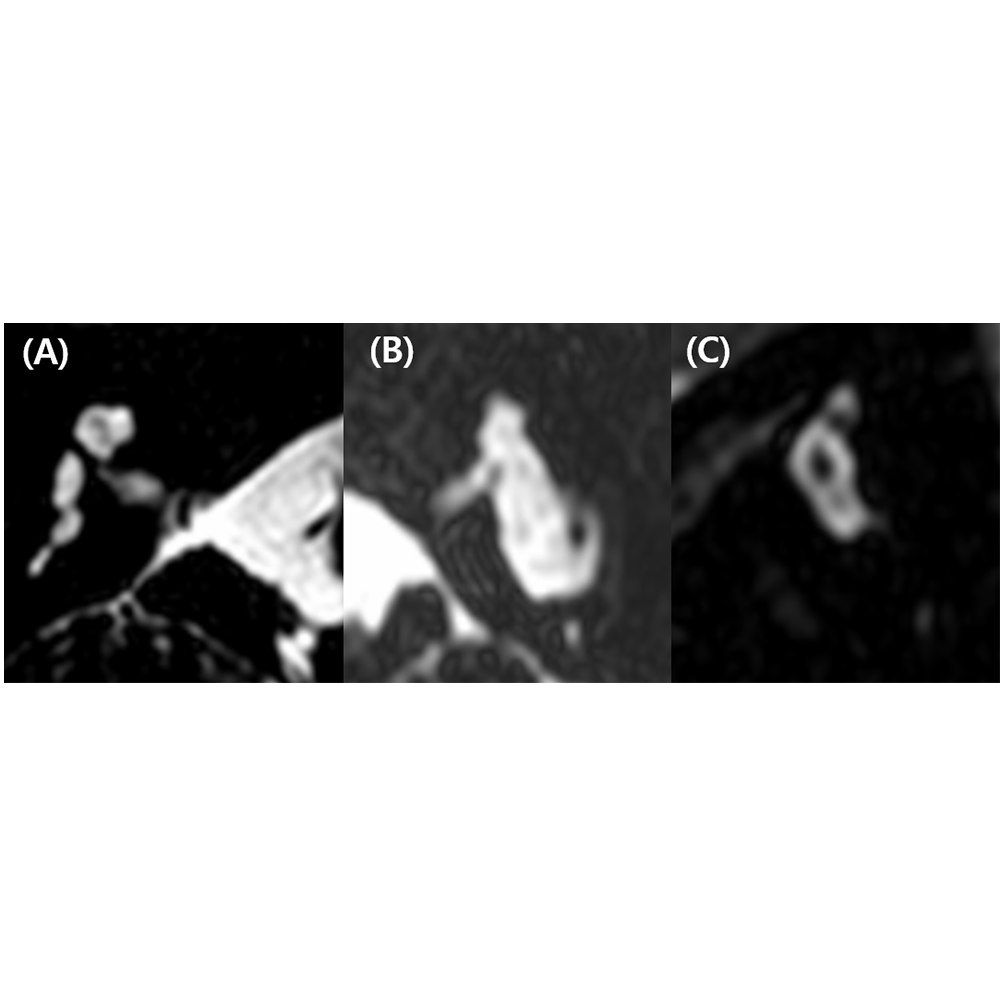

Supplement: Supplementary file 1 [file jcm-07-00515-s001.zip › Supplement 3.tif]

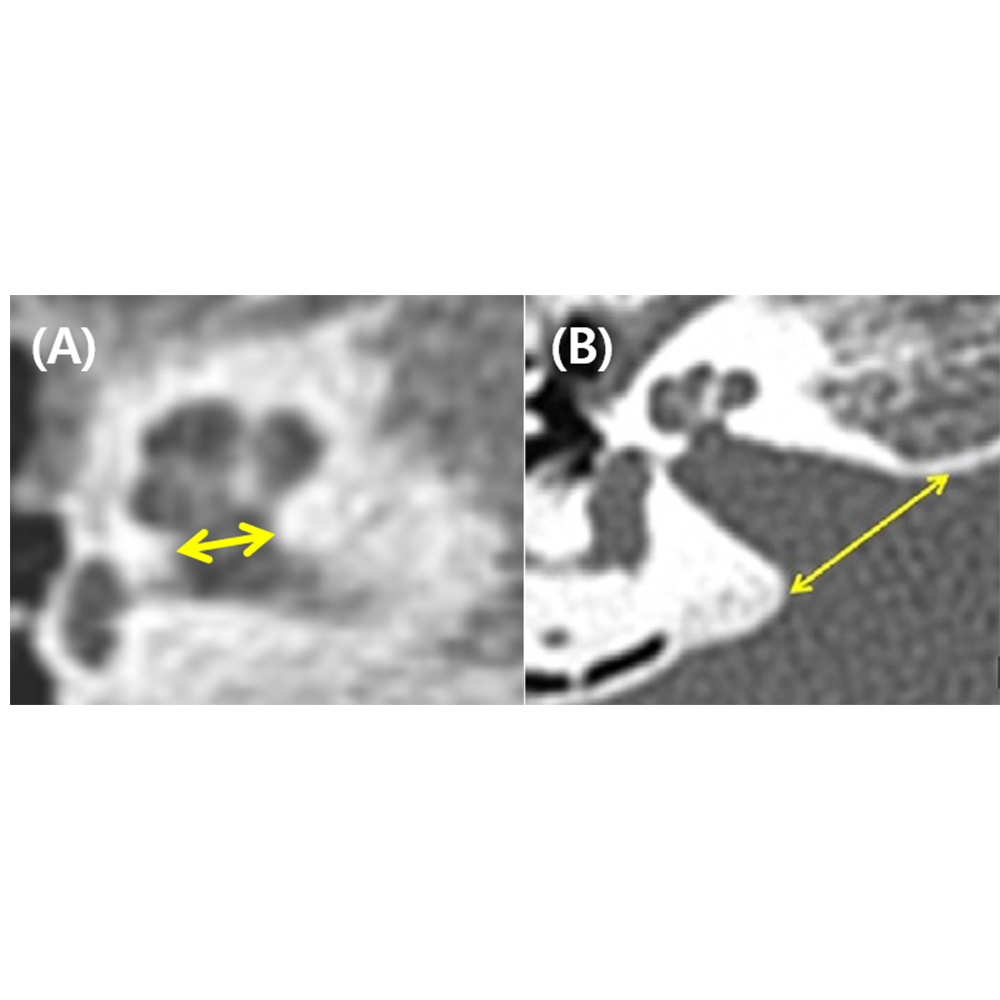

Supplement: Supplementary file 1 [file jcm-07-00515-s001.zip › Supplment 1.tif]
